# Prenylated phenolics from *Morus alba* against MRSA infections as a strategy for wound healing

**DOI:** 10.3389/fphar.2022.1068371

**Published:** 2022-11-30

**Authors:** Gabriela Škovranová, Marie Čulenová, Jakub Treml, Lucia Dzurická, Ivana Marova, Alice Sychrová

**Affiliations:** ^1^ Department of Natural Drugs, Faculty of Pharmacy, Masaryk University, Brno, Czechia; ^2^ Department of Molecular Pharmacy, Faculty of Pharmacy, Masaryk University, Brno, Czechia; ^3^ Institute of Food Science and Biotechnology, Faculty of Chemistry, Brno University of Technology, Brno, Czechia

**Keywords:** antibacterial activity, antimicrobial resistance, kuwanon C, MRSA, mulberry, prenylated phenolics, synergy, wound healing

## Abstract

Antimicrobial resistance is a public health threat and the increasing number of multidrug-resistant bacteria is a major concern worldwide. Common antibiotics are becoming ineffective for skin infections and wounds, making the search for new therapeutic options increasingly urgent. The present study aimed to investigate the antibacterial potential of prenylated phenolics in wound healing. Phenolic compounds isolated from the root bark of *Morus alba* L. were investigated for their antistaphylococcal potential both alone and in combination with commonly used antibiotics. The minimum inhibitory concentration (MIC) and the minimum bactericidal concentration (MBC) were determined by microdilution and agar method. Synergy was investigated using the checkerboard titration technique. Membrane-disrupting activity and efflux pump inhibition were evaluated to describe the potentiating effect. Prenylated phenolics inhibited bacterial growth of methicillin-resistant *Staphylococcus aureus* (MRSA) at lower concentrations (MIC 2–8 μg/ml) than commonly used antibiotics. The combination of active phenolics with kanamycin, oxacillin, and ciprofloxacin resulted in a decrease in the MIC of the antimicrobial agent. Kuwanon C, E, T, morusin, and albafuran C showed synergy (FICi 0.375–0.5) with oxacillin and/or kanamycin. Prenylated phenolics disrupted membrane permeability statistically significantly (from 28 ± 16.48% up to 73 ± 2.83%), and membrane disruption contributes to the complex antibacterial activity against MRSA. In addition, kuwanon C could be considered an efflux pump inhibitor. Despite the antibacterial effect on MRSA and the multiple biological activities, the prenylated phenolics at microbially significant concentrations have a minor effect on human keratinocyte (HaCaT) viability. In conclusion, prenylated phenolics in combination with commonly used antibiotics are promising candidates for the treatment of MRSA infections and wound healing, although further studies are needed.

## 1 Introduction

Wound healing is a complex process of restoring the barrier that protects our bodies from the environment. This process can be affected by wound infections caused by various bacteria such as Staphylococci, the most common commensal inhabitants of human skin (Sychrová et al., 2022). Thus, *Staphylococcus aureus* (SA) has easy access to wounds and is associated with numerous skin and soft tissue conditions, including chronic wound infections (Yokota et al., 2021). Antibiotic treatment of chronic wounds is challenging as the bacteria tend to become resistant when the drugs are used inappropriately for a long time (Tchero et al., 2018). SA infections are difficult to treat due to antibiotic resistance and the high number of toxins and other virulence factors (Cheung et al., 2021). Patients suffering from MRSA infections have a 64% higher risk of death than patients with drug-susceptible infections (WHO, 2021).

In 2017, the World Health Organization published a list of twelve bacterial families that pose the greatest threat and therefore encouraged research and development of new antibiotics for these specific pathogens. MRSA and fluoroquinolone-resistant *Campylobacter* species are listed as high priority and became a major target for many research groups (WHO, 2017). In 2019, the Centers for Disease Control and Prevention divided germs into three categories based on how concerning they are for human health and identified MRSA as a serious threat to watch (CDC, 2019). In 2019, more than 100,000 deaths caused by MRSA were attributed to resistance. Without a coordinated action plan against antimicrobial resistance, many bacteria can become even more deadly than they are today (Murray et al., 2022).

The lack of active antimicrobial agents makes the discovery of new therapeutic compounds urgent. Plants have been a rich source of drugs for self-medication and alternative medicine for various diseases since ancient times. Natural compounds derived from plants have become increasingly attractive for medical treatment due to their availability, potential efficacy, and lack of side effects (Subramani et al., 2017). Extracts from traditional medicine plants or pure compounds in combination with commonly used antibiotics can provide affordable treatment options for bacterial diseases (Cheesman et al., 2017). The resistance of bacterial strains to commonly used antibiotics can be overcome by effectively combining natural compounds with antibiotics, which has synergistic effects (Cheesman et al., 2017). Synergistic interactions allow the combined final effect to be greater than the sum of the individual components. The concept of antimicrobial synergy is based on a principle that results in improved efficacy, reduced toxicity and adverse side effects, increased bioavailability, and lower dosing (van Vuuren and Viljoen, 2011). A key aspect of combination therapy compared to the development of new antibiotic agents is the ability to restore a drug’s efficacy by reducing the development of resistance and reaching clinical use more quickly at lower development costs (Cheesman et al., 2017).

*M. alba* is a traditional Chinese medicine widely used for asthma, fever, diabetes, anemia, edema, as well as analgesic, diuretic, and hypotensive medicine (Čulenová et al., 2020; Choi et al., 2021). The phytochemical profile indicates white mulberry to be high in the content of flavonoids, terpenoids, coumarins, alkaloids (e.g., 1-deoxynojirimycin), and phenolic acids (Polumackanycz et al., 2019; Zhou et al., 2022). Especially, the root bark of *M. alba* is a rich source of flavonoids (e.g., kuwanon C, morusin, sanggenon H), Diels-Alder adducts (DAAs), such as kuwanon G, H, albanol B, then 2-arylbenzofurans (e.g., mulberrofuran A, B, moracin C), and stilbenes (e.g., resveratrol, oxyresveratrol) (Čulenová et al., 2020; Zhu et al., 2021). In general, phenolics and especially flavonoids possess various properties, including antibacterial, anti-inflammatory, and antioxidant activity (Amparo et al., 2020). Prenylation facilitates lipophilicity and increases the efficacy of interaction with membrane and target proteins (Kushwaha et al., 2019). For the antimicrobial activity of flavonoids to Gram-positive bacteria, lipophilicity is a key factor (Yuan et al., 2022). Moreover, prenylated flavonoids play an important role in key signaling pathways in the body (Shi et al., 2021). Due to their diverse biological activities, prenylated flavonoids are considered promising compounds for wound healing (Sychrová et al., 2022).

The present study aimed to evaluate the *in vitro* antibacterial activity of phenolic compounds isolated from the root bark of *M. alba* alone and in combination with commonly used antibiotics as an alternative treatment for MRSA infection and wound healing. Furthermore, we have described the alteration of membrane integrity caused by the active natural compounds as one of the possible mechanisms of antibacterial activity. In comparison to standard inhibitors, the most promising prenylated flavonoid kuwanon C was evaluated for its ability to inhibit efflux pumps. The perspective application of active phenolics in wound healing prompted us to disprove toxicity on HaCaT and to investigate the effects on cell viability. The results of this report identify prenylated phenolics as potent antibacterial agents in wound healing that could be effective in the treatment of multidrug-resistant bacterial infections.

## 2 Material and methods

### 2.1 Natural compounds

The natural compounds were isolated from ethyl acetate and chloroform extracts of the dried root bark of *M. alba* in the Department of Natural Drugs at the Faculty of Pharmacy, Masaryk University, as previously described (Čulenová et al., 2020). The compounds were identified by ultraviolet and infrared spectroscopy, mass spectrometry, and nuclear magnetic resonance analysis, compared with the literature, and provided with a purity of ≥97% for bioactivity measurement. The chemical structures of all tested compounds are listed in Supplementary Table S1.

### 2.2 Antibacterial activity of natural compounds

#### 2.2.1 Bacterial strains and revitalization

Three bacterial strains of methicillin-susceptible *Staphylococcus aureus* (MSSA 3953, MSSA 6188, MSSA 4223) and five MRSA strains (MRSA 4750, MRSA 7109, MRSA 7110, MRSA 7112, MRSA 7113) were acquired from the Czech Collection of Microorganisms at the Faculty of Science, Masaryk University. All strains were stored in a cryoprotective medium at −80°C. Before the experiment, the bacteria were transferred to a Mueller-Hinton agar plate (Sigma-Aldrich, Germany) and cultured at 37°C for 24 h. Then a single colony was transferred to a sterile Mueller-Hinton agar plate and incubated under the same conditions. After 24 h, the bacterial strain was prepared for the experiments.

#### 2.2.2 Antibacterial activity assay

The MIC was determined by the microdilution method using 96-well microtiter plates according to the instructions for MIC determination reported by the European Committee on Antimicrobial Susceptibility Testing (EUCAST, 2000) and in accordance with the guidelines of the Clinical and Laboratory Standards Institute (CLSI, 2015). Twelve natural compounds were tested for their antimicrobial potential against five MRSA and three MSSA strains. The natural compounds were dissolved in dimethyl sulfoxide (DMSO, Sigma-Aldrich), transferred to a microtiter plate and a serial twofold dilution from the highest concentration of 64 μg/ml was performed in a Mueller Hinton Broth (MHB, Sigma-Aldrich). The potential antibacterial activity of DMSO was evaluated separately using three different primary volumes in a well at 100%, 80%, and 32% (v/v), even when the concentration of DMSO in each well with natural compound did not exceed 2.5% (v/v). Standard antibiotics were purchased from Sigma-Aldrich and served as positive controls. Kanamycin sulfate, oxacillin sodium salt, and vancomycin hydrochloride were dissolved in deionized water. Ciprofloxacin was dissolved in deionized water with the addition of 0.1M hydrochloric acid solution (0.2%, v/v), with negligible effects on bacterial growth. The bacterial suspension in MHB was adjusted to 0.5 McFarland using the Densi-La-Meter (Lachema, Brno, Czech Republic) and diluted in MHB (1:2, v/v). The final concentration of bacteria in one well was 5×10^5^ colony forming units per mL (CFU/ml) according to the recommendation of CLSI, 2022. Finally, the microtiter plate was incubated for 18 ± 2 h at 35 ± 1°C. A growth control (inoculated medium) and a sterility control (sterile medium) were included. The MIC was defined as the lowest concentration that inhibited the visible growth of bacteria and resulted in a turbidity reduction of ≥80% compared to the growth control (Novy et al., 2013; Balouiri et al., 2016). Turbidity was measured spectrophotometrically at 600 nm using Omega FLUOR Microplate (Thermo Fisher Scientific, Waltham, MA, United States) and visually after the addition of 10 µL of 1% 2,3,5-triphenyltetrazolium chloride (TTC, Sigma-Aldrich) solution. The measurement was performed in triplicate and the MIC was determined as the median of three values.

#### 2.2.3 Determination of the bactericidal effect

The agar-aliquot subculture method was used to determine MBC, as previously reported (Balouiri et al., 2016). After the determination of the MIC value, the 10 µL aliquots from wells without growth (MIC, 2×MIC, and 4×MIC) were transferred to Mueller Hinton Agar plates with a sterile bacterial loop and incubated at 35°C for 24 h. MBC was set as the lowest concentration that resulted in the growth of no more than 5 colonies, corresponding to a relative decrease in CFU of 99.9% (Balouiri et al., 2016; Gonec et al., 2022).

### 2.3 Synergy testing

The combination of natural compounds with antibiotics was evaluated using the microdilution checkerboard titration technique. Fractional inhibitory concentration (FIC) was determined using 96-well plates with a final bacterial suspension of 5 × 10^5^ CFU/ml in one well. Twofold serial dilution of the natural compound was performed horizontally, followed by vertical twofold dilution of the antibiotic. Each plate contained the MIC of an antibiotic, the MIC of a natural compound, sterility control, and growth control. After incubation for 18 ± 2 h at 35°C, FIC was assessed for each well with ≥80% turbidity reduction compared to the growth control. Then 10 µL of 1% TTC solution was added and the fractional inhibitory concentration index (FICi) was calculated as the sum of each FIC using the following formula:

∑FICi=FICA+FICB

where FICA is the MIC of the antibiotic in combination divided by the MIC of the antibiotic alone. FICB is the MIC of the natural drug in combination divided by the MIC of the natural drug alone. FICi was interpreted for synergy (FICi ≤0.5), partial synergism (FICi >0.5 ≤ 1), indifference (FICi >1 <2), and antagonism (FICi ≥2) (EUCAST, 2000).

### 2.4 Membrane permeability assay

To evaluate the effect of the active natural compounds on the bacterial membrane, the clinical isolate MRSA 7109 was used due to its properties and resistance genes as previously described (Melter et al., 2003). The membrane disruption assay was performed at a subinhibitory concentration (1/4 of the MIC) for all active natural compounds (MIC ≤8 μg/ml) using the Live/Dead solution consisting of the two fluorescent dyes propidium iodide and SYTO9 (Invitrogen, Thermo Fisher Scientific) prepared according to the instructions of the manufacturer of the Live/Dead bacterial viability kit L-7012 (Molecular Probes, Eugene, OR, United States). Propidium iodide is a membrane-impermeable dye that penetrates bacteria with damaged membranes. SYTO9 is a green fluorescent nucleic acid dye that can freely penetrate living and dead bacterial cells. Because of the 60-s interval for each cycle and the tripled amount of each sample, two plates were prepared separately. In the first plate, kuwanon E, C, morusin, and albafuran C were assessed and in the second plate kuwanon U, H, T, and morusinol were measured. Membrane disruption was performed according to Simunovic et al., 2021 with minor modifications. In brief, after 7 h of cultivation, cells in the mid-exponential phase were washed twice with warm phosphate-buffered saline (PBS, Thermo Fisher Scientific) and adjusted to OD_550_ = 0.4. Natural compounds dissolved in DMSO were diluted to MIC concentration with PBS in small Eppendorf tubes. The Live/Dead solution (100 µL) was added to the bacterial suspension in the black microtiter plate (2:1, v/v). SYTO9 fluorescence was measured at λ_ex_ 485 nm and λ_em_ 520 nm at 60-s intervals. After 10 min, 50 µL of the solutions were added to the plate and measured for 60 min under the same conditions. The dead culture (heated at 80°C, 30 min) and the culture treated with DMSO served as controls on each plate. A blank value (Live/Dead solution and natural compound/DMSO in PBS) was obtained for each sample. The relative fluorescence unit (RFU) values for the blank were subtracted from the RFU values for the compound tested. Each sample was measured in triplicate in three separate experiments. The relative destruction of the membrane was evaluated from the average of the last 10 min in a well considering 100% disruption for the heated culture and 0% for the culture treated with DMSO using the formula:

$$\%\;=100-\left(\frac{tested\;compound-dead\;culture}{DMSO\;treated\;culture-dead\;culture}\times 100\right)$$

### 2.5 Efflux pump inhibition assay

The test was performed as previously described by Tintino et al., 2016. Stock solutions at a concentration of 1024 μg/ml were prepared by dissolving ciprofloxacin and norfloxacin (Sigma-Aldrich) in deionized water with 0.1M hydrochloric acid solution (5%, v/v); cyanide *m*-chlorophenyl hydrazone (CCCP) in dilute 96% ethanol (1:1, v/v); and ethidium bromide (EtBr) in deionized water. Reserpine and kuwanon C were dissolved in DMSO. Chlorpromazine (CPZ) 1 mg/ml solution and verapamil 1 mg/ml solution were diluted to 512 μg/ml. All efflux pump inhibitors (EPIs) were purchased from Sigma-Aldrich. Briefly, the MIC was determined as previously described. Efflux pump inhibition of MRSA 7109 was investigated using a subinhibitory concentration (1/8 of the MIC) of standard EPIs and kuwanon C. The bacterial suspension in MHB was adjusted to 0.5 McFarland and diluted to 10^7^ CFU/ml 150 µL of the bacterial suspension, 1/8 of the MIC of EPI and kuwanon C were made up to 1500 µL in Eppendorf tubes with MHB, mixed well, and transferred vertically into a 96-well microtiter plate. A bacterial suspension in sterile MHB (1:10, v/v) served as a control. 100 µL of antibiotic or EtBr was added to the first column (1:1, v/v) and serially diluted so that the concentration of antibiotic and EtBr was between 512 and 0.5 μg/ml. After 18 ± 2 h of incubation at 35°C, the MIC of EtBr or the antibiotic in combination was determined after the addition of 10 µL of 1% TTC solution.

### 2.6 Cell viability assay

HaCaT (Cell Lines Service, Germany) were grown in Dulbecco´s modified Eagle´s medium supplemented with 10% heat-inactivated fetal bovine serum, and a 1% mixture of penicillin/streptomycin/amphotericin B (purchased from Gibco, Thermo Fischer Scientific). Cells were cultured in a humidified atmosphere containing 5% of CO_2_ at 37°C. The assay was performed according to a modified version of the protocol previously reported (Li et al., 2009). Cells were seeded in 96-well flat-bottomed microplates at a density of 25×10^3^ cells per well, attached to the bottom, and grown for 16–24 h. The tested compounds were dissolved in DMSO and diluted in the culture medium. The final concentration of DMSO in the samples was less than 1%. Medium containing 1% DMSO served as vehicle control, exhibiting no cytotoxic effect. 10% DMSO in the medium served as positive control and pure medium served as a negative control. A total of 100 μL of different concentrations (16–0.5 μg/ml) of the samples were added to each well containing the cell culture and incubated for 24 h. Afterward, 20 μL of the solution of 3-(4,5-dimethylthiazol-2-yl)-2,5-diphenyltetrazolium bromide (MTT, Duchefa Biochemie, Haarlem, Netherlands) dissolved in PBS at a concentration of 2.5 mg/ml was added to each well and incubated for 3 h at 37°C. The dark-purple formazan crystals were dissolved in a detergent solution of DMSO and stored in a dark place at room temperature. The microplates were measured spectrophotometrically the next day at 540 nm using the BioTek Epoch Microplate Spectrophotometer (Agilent, Santa Clara, CA, United States). All experiments were performed in triplicate with two separate passages. The viability of cells treated with the samples was calculated as follows:

$$cell\;viability\;\left(\%\right)=\frac{A1}{A2}\times 100$$

where A1 describes the absorbance of cells treated with the tested substance and A2 describes the absorbance of cells incubated with the culture medium.

### 2.7 Statistical analysis

Membrane integrity results were statistically analyzed using IBM SPSS Statistics for Windows, version 26.0 software (IBM, Armonk, NY, United States). Data for the percentage of membrane disruption were plotted as mean ± standard deviation (STDV). Statistical significance between datasets was determined using a Kruskal-Wallis test followed by pairwise comparison with a Bonferroni correction. Data for the percentage of cell viability were plotted as mean ± STDV.

## 3 Results

### 3.1 Antibacterial activity

Before evaluation, the prenylated phenolics were dissolved in a suitable solvent. Considering the lipophilicity of the prenylated compounds, DMSO was chosen. The potential antibacterial activity of DMSO was evaluated with three different initial concentrations so that a twofold serial dilution allowed a wider range of measured concentrations. The MIC of three MSSA and five MRSA strains is described in Table 1. MSSA strains are more sensitive to DMSO than MRSA strains. However, the MIC of DMSO never decreased in value by 8% for MSSA and 12.5% for MRSA. Consequently, the antibacterial activity of six flavonoids, two DAAs, three 2-arylbenzofurans, and one stilbene was evaluated for their ability to inhibit the growth of three strains of MSSA and five clinical isolates of MRSA. Table 2 shows the MICs and MBCs of these natural compounds. To compare activity with standards, the MICs of antibiotics against MSSA and MRSA strains were evaluated and presented in Table 2. Five prenylated flavonoids (kuwanon E, kuwanon U, kuwanon T, kuwanon C, and morusin) showed MICs ranging from 2 to 8 μg/ml and MBCs from 4 to 16 μg/ml against all strains tested. The hydrophilic substitution of the C-3 prenyl group in morusinol reduced the antibacterial potential and showed MBCs ≥64 μg/ml. DAAs (kuwanon H and albafuran C) showed similar antibacterial activity with MICs of 2‒4 and 4‒8 μg/ml, respectively, but kuwanon H showed stronger bactericidal potential in terms of MBC of both compounds (4‒8 μg/ml versus 8–32 μg/ml). In this experiment, we determined the MIC of oxacillin (≥64 μg/ml) against all five MRSA strains and confirmed resistance to methicillin according to breakpoints set by EUCAST (2022), and CLSI (2022). The measured MIC of ciprofloxacin (≥4 μg/ml) against MRSA 7109, MRSA 7110, MRSA 7112, MRSA 7113 confirmed resistance to ciprofloxacin as previously described (Melter et al., 2003). Four strains (MRSA 4750, MRSA 7110, MRSA 7112, and MRSA 7113) showed resistance to kanamycin with MICs ≥128 μg/ml according to the breakpoint (MIC >8 μg/ml) listed by EUCAST (2022). The active natural compounds inhibited the growth of the bacteria at lower concentrations than the commonly used antibiotics compared to the strains resistant to the antibiotic. Both the 2-arylbenzofurans moracin P and moracin O, and the glycoside mulberroside C showed weak antimicrobial activity with MICs and MBCs ≥64 μg/ml. Oxyresveratrol also showed similar results and proved to be inactive against tested staphylococcal strains.

**TABLE 1:** MIC of DMSO designed in % (v/v) against MSSA (*n* = 3) and MRSA (*n* = 5) strains.

Strains	MIC		
	DMSO 100%	DMSO 80%	DMSO 32%
MSSA	12.5	10–20	8–16
MRSA	12.5–25	20	16

DMSO, dimethyl sulfoxide; MIC, minimum inhibitory concentration; MRSA, methicillin-resistant *Staphylococcus aureus*; MSSA, methicillin-susceptible *Staphylococcus aureus*.

**TABLE 2:** MIC/MBC of natural compounds and MIC of antibiotics against MRSA and MSSA strains defined in μg/ml.

Compound	MRSA 4750	MRSA 7109	MRSA 7110	MRSA 7112	MRSA 7113	MSSA 3953	MSSA 6188	MSSA 4223
Kuwanon E	4/8	8/8	8/8	4/8	4/4	4/4	4/4	4/8
Kuwanon U	4/4	8/8	8/8	4/8	4/4	4/8	4/4	4/8
Kuwanon T	8/8	4/8	4/8	8/16	4/16	4/4	4/8	8/8
Kuwanon C	4/8	2/8	4/8	4/8	2/16	2/4	4/8	4/8
Morusin	4/8	4/8	8/8	4/8	4/4	2/4	2/4	4/8
Morusinol	8/≥64	8/≥64	8/≥64	8/≥64	8/≥64	8/≥64	8/≥64	16/≥64
Kuwanon H	2/4	2/8	4/8	2/8	2/8	4/4	2/4	2/8
Albafuran C	8/8	8/16	8/16	8/32	8/16	8/8	4/16	8/16
Moracin O	≥64/>64							
Moracin P	≥64/>64							
Mulberroside C	>64/>64							
Oxyresveratrol	>64/>64							
Oxacillin	16	64	1024	512	512	0.25	1	0.125
Ciprofloxacin	0.125	16	16	8	4	0.125	0.5	0.125
Kanamycin	128	0.5	1024	512	512	0.5	1	1
Vancomycin	1	1	1	1	1	1	1	1

MBC, minimum bactericidal concentration; MIC, minimum inhibitory concentration; MRSA, methicillin-resistant *Staphylococcus aureus*; MSSA, methicillin-susceptible *Staphylococcus aureus*.

### 3.2 Synergistic potential

The active natural compounds (MICs <16 μg/ml) were investigated for their synergistic potential in combination with ciprofloxacin, kanamycin, and oxacillin against MRSA strains resistant to selected antibiotics. The FICi of all combinations are shown in Table 3.

**TABLE 3:** The results for all interactions of natural compounds with antibiotics against MRSA strains.

ATB	NC	FICi				
		MRSA 7109	MRSA 7110	MRSA 7112	MRSA 7113	MRSA 4750
CIPROFLOXACIN	Kuwanon E	PSYN	PSYN	PSYN	IND	—
	Kuwanon U	PSYN	PSYN	IND	IND	—
	Kuwanon T	PSYN	PSYN	IND	IND	—
	Kuwanon C	PSYN	PSYN	IND	IND	—
	Morusin	PSYN	PSYN	PSYN	PSYN	—
	Morusinol	PSYN	PSYN	IND	IND	—
	Kuwanon H	IND	PSYN	PSYN	IND	—
	Albafuran C	PSYN	PSYN	IND	IND	—
KANAMYCIN	Kuwanon E	—	PSYN	**SYN**	PSYN	**SYN**
	Kuwanon U	—	PSYN	PSYN	PSYN	PSYN
	Kuwanon T	—	PSYN	PSYN	PSYN	**SYN**
	Kuwanon C	—	**SYN**	**SYN**	PSYN	**SYN**
	Morusin	—	**SYN**	PSYN	PSYN	PSYN
	Morusinol	—	PSYN	PSYN	PSYN	PSYN
	Kuwanon H	—	PSYN	PSYN	PSYN	PSYN
	Albafuran C	—	**SYN**	PSYN	PSYN	**SYN**
OXACILLIN	Kuwanon E	PSYN	PSYN	PSYN	PSYN	PSYN
	Kuwanon U	PSYN	PSYN	PSYN	PSYN	PSYN
	Kuwanon T	**SYN**	PSYN	PSYN	PSYN	**SYN**
	Kuwanon C	PSYN	PSYN	PSYN	PSYN	**SYN**
	Morusin	PSYN	PSYN	PSYN	PSYN	**SYN**
	Albafuran C	IND	IND	PSYN	IND	**SYN**

ATB, antibiotic; FICi, fractional inhibitory concentration index; IND, indifference; MRSA, methicillin-resistant *Staphylococcus aureus*; NC, natural compound; PSYN, partial synergism; SYN, synergy; (—), not tested.

Four prenylated flavonoids showed synergy in combination with kanamycin against at least one clinical MRSA isolate out of six tested (Supplementary Table S2). Kuwanon C showed synergy against three strains and lowered the MIC of the antibiotic 8-fold. Partial synergism was observed with all other combinations with flavonoids. Two DAAs in combination with aminoglycoside showed partial synergism. However, albafuran C potentiated the effect of kanamycin, and synergy was described for two strains.

Six prenylated phenolics were tested together with oxacillin against five clinical isolates and the results are shown in Supplementary Table S3. Four of the compounds tested (kuwanon C, kuwanon T, morusin, and albafuran C) showed synergy and enhanced the effect of the cell wall-active antibiotic. Partial synergy was described for most combinations with five prenylated flavonoids.

The combinations of eight natural compounds with ciprofloxacin against four MRSA strains are listed in Supplementary Table S4. Partial synergism was assessed for all compounds against at least two MRSA strains. DAAs lowered the MIC of the antibiotic twofold and kuwanon H showed a partially synergistic effect with 1/16 of the MIC. For the other strains, the results were insignificant and indifference occurred. Flavonoids substituted with prenyl and geranyl enhanced the effect of ciprofloxacin and decreased the MIC of the antibiotic by 2–4-fold. Partial synergism of eight tested compounds was observed in at least two MRSA strains (FICi 0.531–1). The rest of the combinations showed insignificant results. Morusin is considered the most effective compound, showing partial synergism in all MRSA strains resistant to ciprofloxacin. Aelenei et al. (2020) described the 64-fold reduction in the MIC of ciprofloxacin and synergism in combination with morusin.

### 3.3 Membrane disruption

**FIGURE 1:**
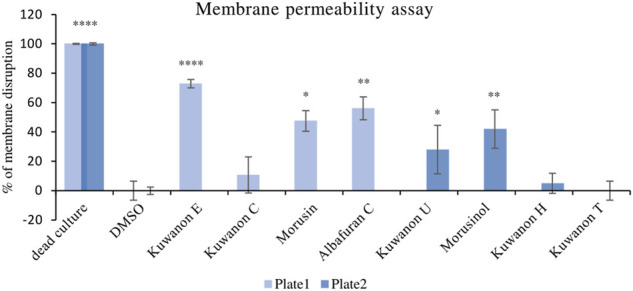
Graphical evaluation of the membrane disruption assay of prenylated phenolics against MRSA 7109.

**FIGURE 2:**
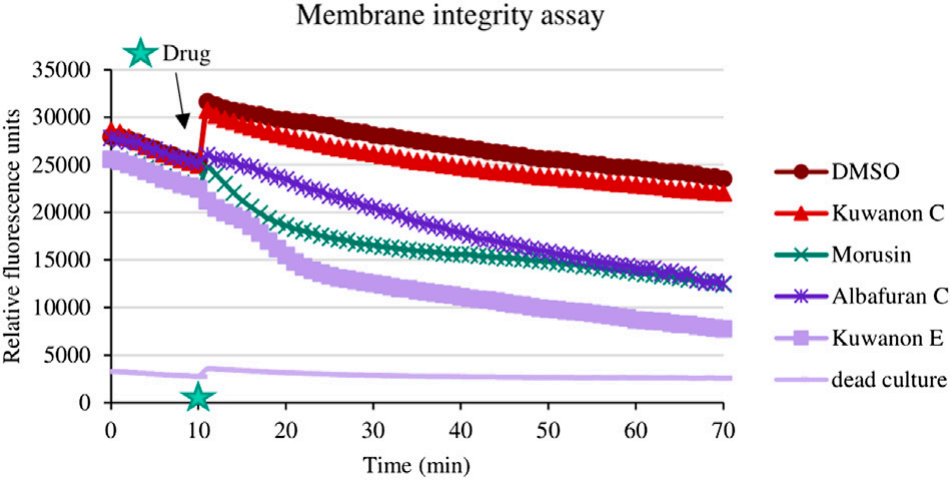
Illustrated effect of natural compounds on membrane integrity during the experiment.

To explain the mechanism of combined activity, eight compounds were screened for disruption of membrane permeabilization. Fluorescence quenching of SYTO9 by propidium iodide was measured at 1/4 of the MIC. The average value of membrane disruption for each compound with the statistical deviation is listed in Table 4 and graphically described in terms of statistical significance in Figure 1. For illustration, the membrane-disrupting effect of four natural compounds on plate 1 during a 70 min measurement is shown in Figure 2. The membrane disruption is described by relative fluorescence units in each minute of the measurement. From the group of six flavonoids and two DAAs, we found five compounds that disrupted membrane permeability in a statistically significant manner (kuwanon E with ****p < 0.0001; morusinol and albafuran C with **p < 0.01; kuwanon U and morusin with *p < 0.05). Kuwanon E decreased membrane permeability by 73 ± 2.83% after 1 h compared to DMSO. Kuwanon U, morusinol, morusin, and albafuran C affected membrane integrity by 28 ± 16.48%, 42 ± 13.11%, 48 ± 7.05%, and 56 ± 7.84%, respectively. Kuwanon C, T, and H showed no significant interaction with membrane potential (11 ± 12.27% for kuwanon C and less for the others).

**TABLE 4:** Membrane disruption (%) of MRSA 7109 caused by natural compounds at 1/4 of the MIC.

Substance	1/4 MIC	% of disruption ± STDV_plate1_ (±STDV_plate2_)
Dead culture	—	100 ± 0.36 (±0.79)
DMSO	—	0 ± 6.48 (±2.55)
Kuwanon E	2	73 ± 2.83
Kuwanon C	0.5	11 ± 12.27
Morusin	1	48 ± 7.05
Albafuran C	2	56 ± 7.84
Kuwanon U	2	28 ± 16.48
Morusinol	2	42 ± 13.11
Kuwanon H	0.5	5 ± 6.82
Kuwanon T	1	0 ± 6.50

DMSO, dimethyl sulfoxide; MIC, minimum inhibitory concentration in μg/ml; MRSA, methicillin-resistant *Staphylococcus aureus*; STDV, standard deviation; (—), not stated.

### 3.4 Efflux pump inhibition

One of the mechanisms by which phenolics combat bacterial resistance to common antibiotics is their ability to inhibit the bacterial efflux system. Efflux pumps are transmembrane proteins designed to protect bacteria from toxins. They expel toxic compounds from the cell so that they are no longer available for their inhibitory action. They are divided into five families that differ in their structure, the substrates they affect, and the energy source. These multidrug transporters are also found on SA and confer resistance to various chemicals, including quaternary ammonium compounds, fluoroquinolones, macrolides, and tetracyclines (Varela and Kumar, 2013).

**FIGURE 3:**
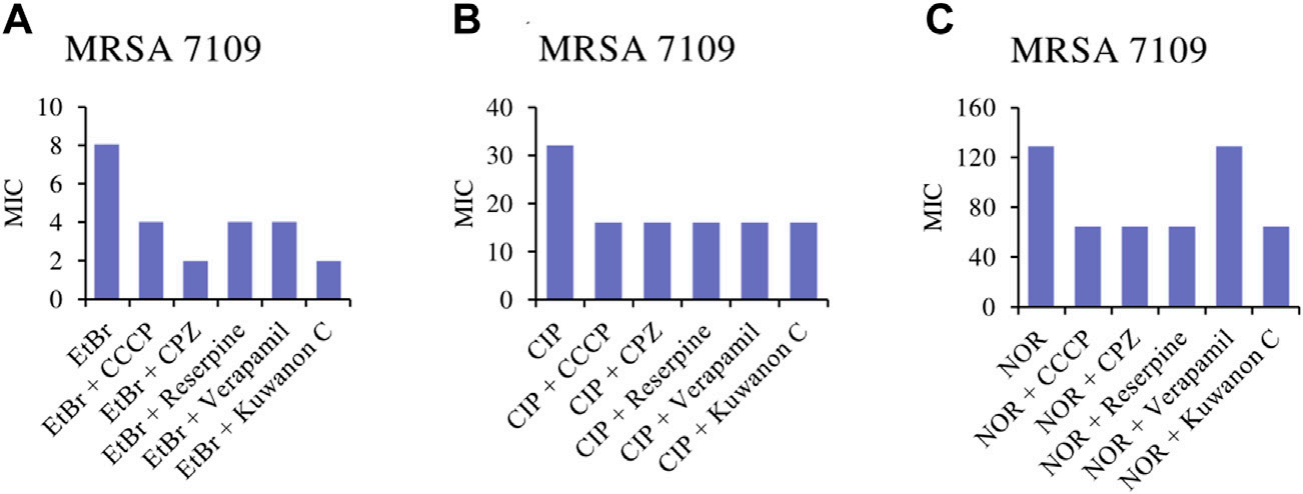
Minimum inhibitory concentrations (MICs) of **(A)** EtBr, ethidium bromide; **(B)** CIP, ciprofloxacin and **(C)** NOR, norfloxacin alone, and in combination with the standard inhibitor CCCP, carbonyl cyanide *m*-chlorophenylhydrazone; CPZ, chlorpromazine; reserpine; verapamil and kuwanon C against MRSA 7109 shown in μg/ml.

To assess whether kuwanon C is a potential EPI, a comparative study was conducted. The MICs of the two antibiotics, ciprofloxacin and norfloxacin, and also EtBr were compared alone as well as in combination with standard EPIs and kuwanon C, as EtBr is a substrate for many multidrug-resistant pumps. The MICs of EPIs, antibiotics, and kuwanon C against MRSA 7109 alone are shown in Table 5. According to the result, 1/8 of the MIC was evaluated in combination with a substrate for multidrug-resistant efflux pumps and two fluoroquinolones. The effect of CCCP, CPZ, verapamil, reserpine, and kuwanon C on the MIC of EtBr, ciprofloxacin, and norfloxacin is shown in Figure 3. Standard EPIs such as CCCP, CPZ, verapamil, reserpine, and the tested kuwanon C lowered the MIC of EtBr 2‒4-fold and showed synergistic effects. In combination with antibiotics, the MIC was lowered 2-fold. The enhancement of the antibacterial activity of antibiotics can be explained by the inhibited extrusion of antimicrobial substances by efflux pumps, which concerns the activity of EPI in combination with the known substrate EtBr.

**TABLE 5:** MIC (µg/ml) of EtBr, standard EPIs, antibiotics, and kuwanon C against MRSA 7109.

Substance	MIC
CCCP	2
CPZ	64
Reserpine	>128
Verapamil	>128
EtBr	8
Kuwanon C	2
Ciprofloxacin	32
Norfloxacin	64

CCCP, cyanide *m*-chlorophenyl-hydrazone; CPZ, chlorpromazine; EtBr, ethidium bromide; MIC, minimum inhibitory concentration; MRSA, methicillin-resistant *Staphylococcus aureus*.

### 3.5 Cell viability

**FIGURE 4:**
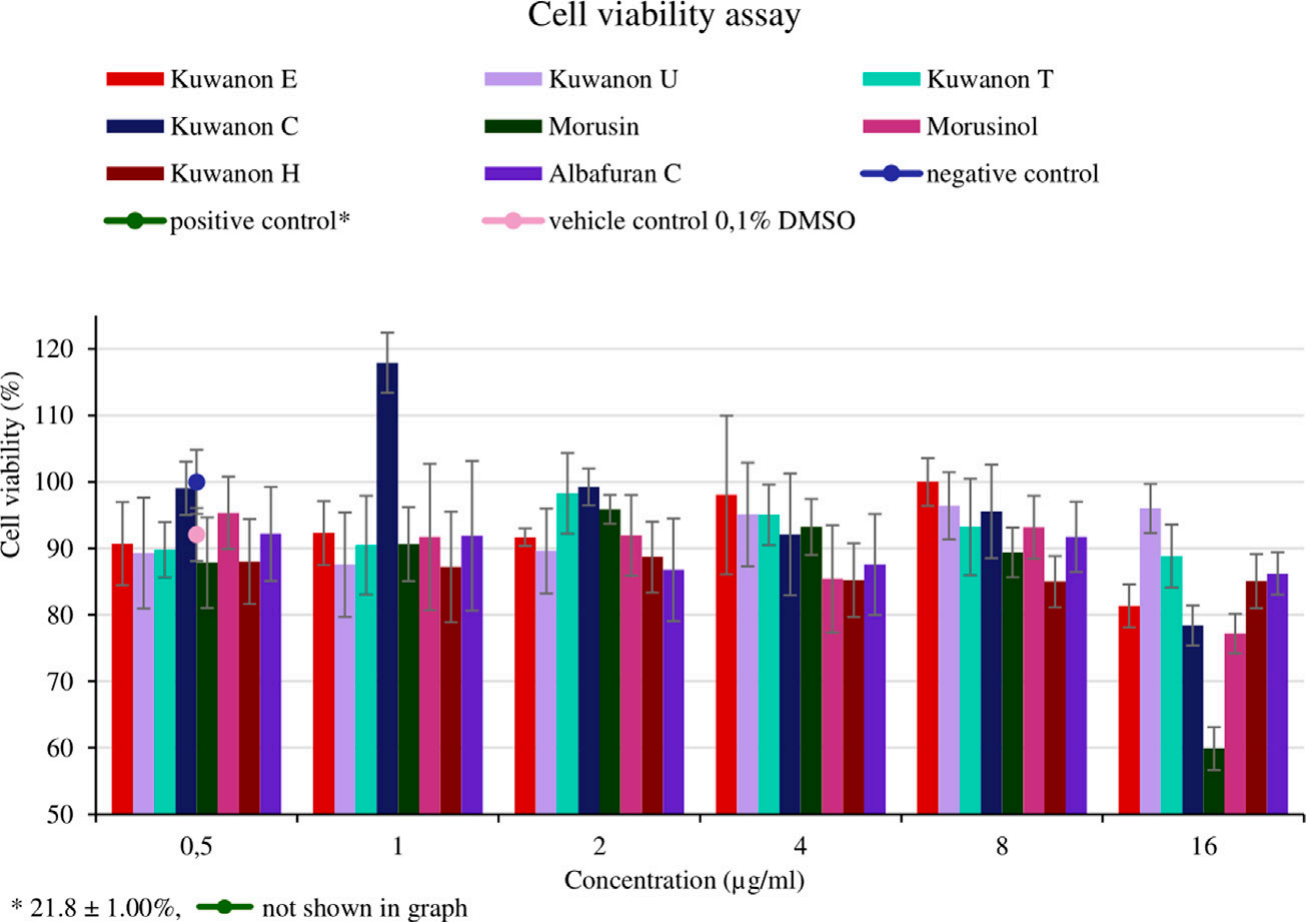
Cell viability of human keratinocytes (HaCaT) treated with natural compounds at different concentrations (µg/ml).

For the application of natural compounds in wound healing, the effect on the viability of HaCaT should be determined. The assay aimed to determine the concentration that does not affect cell viability and to compare it with the MIC of each active compound. Cells from two different passages were treated with eight compounds at five concentrations (16, 8, 4, 2, and 1 μg/ml). The mean percentage of cell viability with standard deviation for each compound is shown in Figure 4. Morusin was the most toxic compound, showing cell viability of 59.9 ± 3.23% at the highest concentration tested. The MICs against bacteria ranged from 2‒8 μg/ml and these concentrations did not affect cell viability (>87%). HaCaT treated with seven other compounds at a concentration of 16 μg/ml showed the viability of more than 77.2 ± 2.96%. Prenylated flavonoids and DAAs at concentrations equivalent to the MICs against MRSA and MSSA strains did not affect cell viability and are considered non-toxic for HaCaT.

## 4 Discussion

Normal skin maintains its integrity and protects the underlying tissue from skin inhabitants (Maheswary et al., 2021). Alteration of the skin barrier can lead to pathological conditions such as skin inflammation, skin infection, allergic reactions, the formation of superficial skin tumors, or a delay in skin healing (Leonel et al., 2019). Wound healing is a systematic response of an organism to the physical destruction of a tissue or organ. The healing process involves the physiological stabilization of the whole organism and the restoration of homeostasis, which can be achieved through scar formation and regeneration (Gurtner et al., 2008). The typical mammalian response to injury can be divided into three distinct phases: inflammation, new tissue formation, and remodeling (Gurtner et al., 2008).

The wound microenvironment plays an important role in the healing process, highlighting the particular importance of microbes (Scalise et al., 2015). In acute wounds, Gram-positive bacteria predominate over Gram-negative bacteria. Chronic wounds are colonized by SA and *Staphylococcus epidermidis* as well as Gram-negative bacteria such as *Escherichia coli*, *Proteus mirabilis*, *Pseudomonas aeruginosa*, *Enterobacter* species, and *Morganella* species (Maheswary et al., 2021). In addition, the microorganisms tend to form a complex, cross‒species structure‒the biofilm‒which is difficult to eradicate by the host immune system and resistant to conventional treatment strategies (Secor et al., 2011).

Natural resources represent an enormous source for the search for new antimicrobial agents. Pure compounds isolated or even synthetically modified from an active template can lead to effective agents for the treatment of various human diseases (Newman and Cragg, 2020). Natural products and their derivatives account for more than 30% of all new molecular agents approved by the US Food and Drug Administration. Despite declining trends since the 1990s, plant compounds account for a quarter, as much as from microbial sources (Patridge et al., 2016).

*M. alba* is a rich source of flavonoids (including prenylated flavonoids, chalcones, and anthocyanins) and various secondary metabolites such as DAAs, terpenoids, alkaloids, phenolic acids, stilbenoids, and coumarins (Chan et al., 2016; He et al., 2018). Leaves, fruits, twigs, and root bark are used in traditional Chinese medicine to prevent and treat numerous diseases (He et al., 2018). Several studies showed the antibacterial potential of mulberry compounds (Wu et al., 2019; Čulenová et al., 2020; Zhu et al., 2021) as well as anti-inflammatory (Rivière et al., 2014; Čulenová et al., 2020) and antioxidant abilities (Khan et al., 2013; Chan et al., 2016).

In this context, we investigated phenolic compounds isolated from the root bark of *M. alba* as potential agents important for the treatment of MRSA infections and wound healing. Due to the lipophilic nature of the prenylated phenolics, the possible antibacterial effect of DMSO as the solvent was tested in the first step. The MIC of DMSO did not decrease by 8% in MSSA and by 12.5% in MRSA. Based on these results, we can conclude that DMSO has no significant effect on cell viability, even in a well with the highest concentration of the natural compound, where the concentration of DMSO is 2.5% (v/v). We demonstrated the antibacterial activity of twelve phenolics against MSSA and MRSA, both alone and in combination with antibiotics. The flavonoids kuwanon E, kuwanon U, kuwanon T, kuwanon C, and morusin inhibited the growth of MRSA at lower concentrations than standard antibiotics to which resistance has been described. The study showed no significant change in activity depending on substitution with free prenyl (morusin), diprenyl (kuwanon T, kuwanon C), or geranyl (kuwanon E and kuwanon U) moieties with respect to the MICs of prenyl- (2‒8 μg/ml), diprenyl- (2‒8 μg/ml), and geranyl- (4‒8 μg/ml) substituted flavonoids. Cyclization of the C8 prenyl with the C7 hydroxyl group in morusin may decrease the antibacterial activity of the flavone compared to free prenylated flavone‒kuwanon C (Xie et al., 2015). In general, the anti-MRSA activity of flavonoids is based on substitution with hydroxyl groups at C5, C7, and C4’. Dihydroxylation of C2’, C4’ or C2’, C6’ and substitution with an aliphatic chain at C6 or C8 of flavanone increase the antibacterial activity (Xie et al., 2015). However, the presence of a free prenyl group is critical for antibacterial activity. C3 modification of the prenyl moiety with a hydroxyl group (e.g., morusinol) decreased bactericidal activity (MBC ≥64 μg/ml) compared to morusin with free prenyl. According to the MBC/MIC ratio, active phenolic compounds have MBC no higher than four times the MIC and are considered bactericidal (French, 2006).

Prenylated flavonoids and DAAs showed not only strong antibacterial activity but also promising antibiotic-potentiating effects. The antibiotics evaluated for combined effects belong to different classes and mechanisms of action (kanamycin‒aminoglycosides: inhibition of protein synthesis; oxacillin‒beta-lactams: inhibition of cell wall synthesis; ciprofloxacin‒fluoroquinolones: inhibition of DNA synthesis) (Kohanski et al., 2010). The antibacterial activity of DAAs and their synergistic potential with aminoglycosides have been reported previously. Albanin G (syn. kuwanon H) lowered the MIC of amikacin 4–16-fold against ten MRSA strains and showed synergy at 80% (*n* = 10). Multicaulisin and sanggenon G also showed synergistic potential in combination with aminoglycosides such as amikacin, etimicin, and gentamicin at varying rates (40%–80% of ten MRSA strains tested) (Zuo et al., 2019). Kuwanon G acted synergistically with gentamicin against MRSA and partial synergism was noted against MSSA (Aelenei et al., 2020). Morusin in combination with gentamicin lowered the MIC of the antibiotic 16-fold (Aelenei et al., 2020). Partial synergism was described when morusin was combined with etimicin and amikacin in 70% and 80% of strains (*n* = 10), respectively, and synergy for the remainder. In combination with kuwanon E, synergy was shown in 70% and 80% of strains (Zuo et al., 2019). The present study supports the results of the synergistic potential of morusin and kuwanon E with aminoglycosides. In addition, the antibacterial activity of kuwanon T has been described, but the synergistic potential has not been reported to our knowledge. Kuwanon T and kuwanon C showed synergism in combination with kanamycin and oxacillin against at least one strain of MRSA and partial synergism with the others. Morusin and albafuran C showed synergistic effects in combination with oxacillin. Morusin also reversed the oxacillin resistance of MRSA by lowering its MIC to 1 ug/ml when combined (Aelenei et al., 2020). All prenylated phenolics potentiated the activity of the membrane-targeted antibiotic oxacillin by 2‒8-fold and showed synergistic and/or partially synergistic activity against all strains tested. In combination with ciprofloxacin, partial synergism was shown with all eight compounds. Anti-MRSA activity in combination has been reported previously, and kuwanon H showed synergy with levofloxacin. Synergy was demonstrated in 20% of the MRSA strains tested, and values of 0.5 > FICi≤1.5 were obtained for the rest of the strains, which is consistent with our results (Zuo et al., 2019). All compounds showed partial synergistic interaction, with morusin identified as the most potent compound. Resistance of MRSA strains to ciprofloxacin was not abolished in combination with the tested compounds with respect to the breakpoint of CLSI (2022) (R ≥ 4 μg/ml). The mechanism of resistance to fluoroquinolones is based on a mutation in the *grlA*/*grlB* genes encoding topoisomerase IV and the *gyrA*/*gyrB* gene encoding DNA gyrase. These changes decrease the affinity of the drug. Another mechanism is attributed to efflux pumps (Hassanzadeh et al., 2017). As mentioned earlier, ciprofloxacin is active inside the cell. Therefore, the natural compounds could interact with the integrity of the cell, increasing the uptake of the antibiotic inside the cell and decreasing the MIC. Another explanation could be the ability of the drug to inhibit efflux pumps.

To determine whether prenylated flavonoids inhibit the extrusion of an antibiotic from the cell, we needed to describe the disruptive effect on a membrane. In this context, we tested all active compounds. There is a correlation between the percentage of membrane disruption and the concentration tested. Morusin, albafuran C, kuwanon U, E, and morusinol interacted with the membrane to a statistically significant extent because the concentrations tested (1/4 of the MIC) were ≤2 μg/ml. We could predict that membrane disruption may be one of the mechanisms of natural compounds. Aelenei et al. (2020) described morusin as a membrane-active flavone that showed disruption of proton motive force and cytoplasmic membrane permeability of MRSA. Membrane-active compounds have the potential for future antibiotic development because it is difficult for bacteria to develop resistance to membrane-targeted drugs (Wu et al., 2019). Kuwanon C, T, and H did not significantly alter membrane permeability, so we performed an efflux pump inhibition assay.

Since efflux pumps are responsible for expelling toxic compounds into the extracellular space, inhibiting efflux would increase the concentration of the antibacterial agent inside. The gene *mrsA* encodes an efflux pump of the ABC transporter family that confers resistance to macrolides and lincosamides (Figueredo et al., 2021). Resistance to tetracyclines in SA is conferred by the efflux pump Tet(K). The chromosomally localized gene *norA* is responsible for relatively high resistance to various hydrophilic quinolones (e.g., ciprofloxacin, norfloxacin) and chloramphenicol, and overexpression of *norA* has been demonstrated in clinical isolates (Köhler et al., 1999). Several efflux pump inhibitors have been discovered. CCCP is an energy uncoupler that collapses membrane energy involved in the efflux process (Malléa et al., 2002). CPZ is a competitive inhibitor that binds to the same target or near the binding site of the antibiotic substrate (Tintino et al., 2016). Verapamil is known to inhibit norA pumps but it is also a calcium channel blocker (Holler et al., 2012). Natural compounds such as quercetin (dos Santos et al., 2021), kaempferol rhamnoside, and reserpine showed potential inhibition of the norA efflux pump in SA (Holler et al., 2012). In addition, efflux pump inhibition has been described in MRSA for kuwanon O isolated from *M. alba* (Zhu et al., 2021). Prenylated flavonoids stay at the forefront of our attention, with kuwanon C, and kuwanon T being the most attractive and prospective compounds. However, the isolated amount of kuwanon T was limited, so kuwanon C was chosen to assess its potential to inhibit the efflux pumps. MRSA 7109, a resistant strain that poses gene *msr*A (Melter et al., 2003), was not tested for detection of the *norA* gene. However, we investigated the potential of standard efflux pump inhibitors to increase the activity of EtBr and decrease its MIC. EtBr is a substrate for many multidrug-resistant pumps. Considering the 2‒4-fold reduction in the MIC of EtBr, we combined standard EPIs with two antibiotics classified as fluoroquinolones. The higher efficacy of ciprofloxacin and norfloxacin can be explained by the inhibitory effect of the selected EPI, which resulted in a twofold reduction in MIC. Fortunately, kuwanon C showed similar or even better activity than three EPIs (CCCP, verapamil, and reserpine) at the subinhibitory concentration (1/8 of the MIC), as the MIC of EtBr decreased 4-fold and 2-fold in combination with ciprofloxacin and norfloxacin, respectively. Therefore, we could consider kuwanon C as a potential inhibitor of efflux pumps in multidrug-resistant clinical strain MRSA 7109.

Phenolics have been mentioned as promising therapeutic agents in wound healing. Their multiple activities involved in wound healing are crucial, as is their low toxicity to human cells. HaCaT are the most abundant cells in the epidermis and essential components of the healing process. They constantly respond to bacterial stimuli and participate in innate immunity by providing a physical barrier to the external environment. The production of inflammatory cytokines by HaCaT is an essential step in a normal immune response and dysregulation can lead to chronic inflammation (Secor et al., 2011). Prenylated phenolics did not alter the viability of HaCaT at concentrations equivalent to the MICs and could be considered promising compounds for the treatment of skin infections and wound healing.

## 5 Conclusion

Prenylated phenolics from the root bark of *M. alba* are promising compounds for the treatment of MRSA infections and microbially infected wounds. The present study demonstrates the antimicrobial activity of six prenylated flavonoids and two DAAs when they inhibited the growth of MRSA at lower concentrations than standard antibiotics. In addition, kuwanon C, E, T, morusin, and albafuran C showed synergistic effects when combined with oxacillin and/or kanamycin against MRSA isolates. Insight into the mode of action suggests that membrane disruption plays an important role in the complex antibacterial activity of prenylated phenolics. Nevertheless, kuwanon C could be considered a potent efflux pump inhibitor. When comparing the antibacterial potential of prenylated phenolics with cytotoxicity to human cells, it was found that the effective MIC against MRSA did not affect the viability of HaCaT cells. Overall, these results contribute to the perspective strategies to reduce the consumption of commonly used antibiotics and to reduce the occurrence of multidrug-resistant strains of SA. In addition to antibacterial activity, prenylated flavonoids provide various biological properties beneficial for wound healing, such as antioxidant or anti-inflammatory activities. The prospective qualities of prenylated flavonoids and the results of this report may be useful for the development of new anti-infective drugs effective in wound healing and warrant further studies of pharmacological efficacy *in vivo*.

## Data Availability

The original contributions presented in the study are included in the article/Supplementary Material, further inquiries can be directed to the corresponding authors.

## Abbreviations

CCCP, cyanide m-chlorophenyl-hydrazone; CFU, colony-forming units; CLSI, Clinical and Laboratory Standards Institute; CPZ, chlorpromazine; DAA, Diels-Alder adduct; DMSO, dimethyl sulfoxide; EPI, efflux pump inhibitor; EtBr, ethidium bromide; EUCAST, European Committee on Antimicrobial Susceptibility Testing; FIC, fractional inhibitory concentration; FICi, FIC index; HaCaT, human keratinocytes; *M. alba*, *Morus alba*; MBC, minimum bactericidal concentration; MIC, minimum inhibitory concentration; MRSA, methicillin-resistant *Staphylococcus aureus*; MSSA, methicillin-susceptible *Staphylococcus aureus*; MTT, 3-(4,5-dimethylthiazol-2-yl)-2,5-diphenyltetrazolium bromide; PBS, phosphate-buffered saline; SA, *Staphylococcus aureus*; STDV, standard deviation; TTC, 2,3,5-triphenyltetrazolium chloride solution.
