# Supplementary material for: Prenylated phenolics from Morus alba against MRSA infections as a strategy for wound healing
Source: Front Pharmacol. 2022 Nov 30;13:1068371. doi: 10.3389/fphar.2022.1068371 (PMC9747775; doi:10.3389/fphar.2022.1068371)
Supplement: Supplementary file 1 [file DataSheet1.docx]

Supplementary Material

# Supplementary Figures and Tables

## Supplementary Tables

Table S1 Chemical structures of twelve natural compounds from root bark of *Morus alba*

| Prenylated flavonoids: | | | |
| --- | --- | --- | --- |
|   Kuwanon E |   Kuwanon T | |   Kuwanon C |
|   Kuwanon U |   Morusin | |   Morusinol |
| Diels-Alder adducts: | | | |
|   Kuwanon H | |   Albafuran C | |
| 2-arylbenzofurans: | | | **Glykoside of 2-arylbenzofuran:** |
|   Moracin O |   Moracin P | |   Mulberroside C |
| Stilbene: | | | |
|   Oxyresveratrol | | | |

Table S2 Natural compounds in combination with kanamycin against MRSA strains

| **NC** | **Strain** | **MIC** | | | | **FICi** | **Effect** |
| --- | --- | --- | --- | --- | --- | --- | --- |
|  |  | **NC** | **ATB** | **NC comb** | **ATB comb** |  |  |
| **Kuwanon E** | MRSA 7110 | 8 | 1024 | 0.5 | 512 | 0.563 | PSYN |
|  | MRSA 7112 | 4 | 512 | 1 | 128 | 0.5 | **SYN** |
|  | MRSA 7113 | 4 | 512 | 2 | 32 | 0.563 | PSYN |
|  | MRSA 4750 | 4 | 128 | 1 | 32 | 0.5 | **SYN** |
| **Kuwanon U** | MRSA 7110 | 8 | 1024 | 1 | 512 | 0.625 | PSYN |
|  | MRSA 7112 | 4 | 512 | 1 | 256 | 0.75 | PSYN |
|  | MRSA 7113 | 4 | 512 | 2 | 256 | 1 | PSYN |
|  | MRSA 4750 | 4 | 128 | 2 | 32 | 0.75 | PSYN |
| **Morusinol** | MRSA 7110 | 8 | 1024 | 1 | 512 | 0.625 | PSYN |
|  | MRSA 7112 | 8 | 512 | 4 | 128 | 0.75 | PSYN |
|  | MRSA 7113 | 8 | 512 | 4 | 128 | 0.75 | PSYN |
|  | MRSA 4750 | 8 | 128 | 4 | 16 | 0.625 | PSYN |
| **Kuwanon T** | MRSA 7110 | 4 | 1024 | 0.5 | 512 | 0.625 | PSYN |
|  | MRSA 7112 | 8 | 512 | 2.4 | 256 | 1 | PSYN |
|  | MRSA 7113 | 4 | 512 | 2 | 256 | 1 | PSYN |
|  | MRSA 4750 | 8 | 128 | 2 | 32 | 0.5 | **SYN** |
| **Kuwanon C** | MRSA 7110 | 4 | 1024 | 1 | 128 | 0.375 | **SYN** |
|  | MRSA 7112 | 4 | 512 | 1 | 128 | 0.5 | **SYN** |
|  | MRSA 7113 | 2 | 512 | 1 | 256 | 1 | PSYN |
|  | MRSA 4750 | 4 | 128 | 1 | 16 | 0.375 | **SYN** |
| **Morusin** | MRSA 7110 | 8 | 1024 | 2 | 256 | 0.5 | **SYN** |
|  | MRSA 7112 | 4 | 512 | 2 | 128 | 0.75 | PSYN |
|  | MRSA 7113 | 4 | 512 | 2 | 64 | 0.625 | PSYN |
|  | MRSA 4750 | 4 | 128 | 0.5 | 64 | 0.625 | PSYN |
| **Kuwanon H** | MRSA 7110 | 4 | 1024 | 2 | 256 | 0.75 | PSYN |
|  | MRSA 7112 | 2 | 512 | 1 | 256 | 1 | PSYN |
|  | MRSA 7113 | 2 | 512 | 0.25 | 256 | 0.625 | PSYN |
|  | MRSA 4750 | 2 | 128 | 1 | 32 | 0.75 | PSYN |
| **Albafuran C** | MRSA 7110 | 8 | 1024 | 2 | 256 | 0.5 | **SYN** |
|  | MRSA 7112 | 8 | 512 | 4 | 128 | 0.75 | PSYN |
|  | MRSA 7113 | 8 | 512 | 4 | 128 | 0.75 | PSYN |
|  | MRSA 4750 | 8 | 128 | 2 | 32 | 0.5 | **SYN** |

ATB, antibiotic; comb, in combination; FICi, fractional inhibitory concentration index; MIC, minimum inhibitory concentration in µg/ml; MRSA, methicillin-resistant *Staphylococcus aureus*; NC, natural compound; PSYN, partial synergism; SYN, synergy

Table S3 Combination of natural compounds with oxacillin against MRSA strains

| **NC** | **Strain** | **MIC** | | | | **FICi** | **Effect** |
| --- | --- | --- | --- | --- | --- | --- | --- |
|  |  | **NC** | **ATB** | **NC comb** | **ATB comb** |  |  |
| **Kuwanon E** | MRSA 7109 | 8 | 64 | 4 | 8 | 0.625 | PSYN |
|  | MRSA 7110 | 8 | 1024 | 4 | 128 | 0.625 | PSYN |
|  | MRSA 7112 | 4 | 512 | 2 | 64 | 0.625 | PSYN |
|  | MRSA 7113 | 4 | 256 | 2 | 64 | 0.75 | PSYN |
|  | MRSA 4750 | 4 | 64 | 2 | 16 | 0.75 | PSYN |
| **Kuwanon U** | MRSA 7109 | 8 | 64 | 4 | 16 | 0.75 | PSYN |
|  | MRSA 7110 | 8 | 1024 | 0.5 | 512 | 0.563 | PSYN |
|  | MRSA 7112 | 4 | 512 | 2 | 128 | 0.75 | PSYN |
|  | MRSA 7113 | 4 | 256 | 2 | 64 | 0.75 | PSYN |
|  | MRSA 4750 | 4 | 64 | 2 | 16 | 0.75 | PSYN |
| **Kuwanon T** | MRSA 7109 | 8 | 128 | 2 | 32 | 0.5 | **SYN** |
|  | MRSA 7110 | 8 | 1024 | 2 | 512 | 0.75 | PSYN |
|  | MRSA 7112 | 4 | 512 | 2 | 256 | 1 | PSYN |
|  | MRSA 7113 | 4 | 256 | 0.125 | 128 | 0.531 | PSYN |
|  | MRSA 4750 | 4 | 64 | 1 | 8 | 0.375 | **SYN** |
| **Kuwanon C** | MRSA 7109 | 2 | 128 | 1 | 64 | 1 | PSYN |
|  | MRSA 7110 | 4 | 1024 | 2 | 512 | 1 | PSYN |
|  | MRSA 7112 | 4 | 512 | 2 | 256 | 1 | PSYN |
|  | MRSA 7113 | 2 | 256 | 0.125 | 128 | 0.563 | PSYN |
|  | MRSA 4750 | 2 | 64 | 0.5 | 16 | 0.5 | **SYN** |
| **Morusin** | MRSA 7109 | 4 | 128 | 2 | 64 | 1 | PSYN |
|  | MRSA 7110 | 4 | 1024 | 0.25 | 512 | 0.563 | PSYN |
|  | MRSA 7112 | 4 | 512 | 1 | 256 | 0.75 | PSYN |
|  | MRSA 7113 | 4 | 256 | 1 | 128 | 0.75 | PSYN |
|  | MRSA 4750 | 2 | 64 | 0.5 | 8 | 0.375 | **SYN** |
| **Albafuran C** | MRSA 7109 | 8 | 128 | 0.25 | 128 | 1.031 | IND |
|  | MRSA 7110 | 8 | 1024 | 0.25 | 1024 | 1.031 | IND |
|  | MRSA 7112 | 8 | 512 | 4 | 256 | 1 | PSYN |
|  | MRSA 7113 | 8 | 256 | 8 | 16 | 1.063 | IND |
|  | MRSA 4750 | 8 | 64 | 2 | 16 | 0.5 | **SYN** |

ATB, antibiotic; comb, in combination; FICi, fractional inhibitory concentration index; IND, indifference; MIC, minimum inhibitory concentration in µg/ml, MRSA, methicillin-resistant *Staphylococcus aureus*; NC, natural compound; PSYN, partial synergism; SYN, synergy

Table S4 Natural compounds in combination with ciprofloxacin against MRSA strains

| **NC** | **Strain** | **MIC** | | | | **FICi** | **Effect** |
| --- | --- | --- | --- | --- | --- | --- | --- |
|  |  | **NC** | **ATB** | **NC comb** | **ATB comb** |  |  |
| **Kuwanon E** | MRSA 7109 | 8 | 16 | 4 | 4 | 0.75 | PSYN |
|  | MRSA 7110 | 8 | 16 | 0.5 | 8 | 0.563 | PSYN |
|  | MRSA 7112 | 4 | 8 | 0.25 | 4 | 0.563 | PSYN |
|  | MRSA 7113 | 4 | 4 | 4 | 0.5 | 1.125 | IND |
| **Kuwanon U** | MRSA 7109 | 8 | 16 | 4 | 8 | 1 | PSYN |
|  | MRSA 7110 | 8 | 16 | 1 | 8 | 0.625 | PSYN |
|  | MRSA 7112 | 4 | 8 | 4 | 0.5 | 1.063 | IND |
|  | MRSA 7113 | 4 | 4 | 0.125 | 4 | 1.031 | IND |
| **Morusinol** | MRSA 7109 | 8 | 16 | 4 | 4 | 0.75 | PSYN |
|  | MRSA 7110 | 8 | 16 | 0.5 | 8 | 0.563 | PSYN |
|  | MRSA 7112 | 8 | 8 | 0.25 | 8 | 1.031 | IND |
|  | MRSA 7113 | 8 | 4 | 8 | 0.5 | 1.125 | IND |
| **Kuwanon T** | MRSA 7109 | 4 | 16 | 2 | 4 | 0.75 | PSYN |
|  | MRSA 7110 | 4 | 16 | 2 | 4 | 0.75 | PSYN |
|  | MRSA 7112 | 8 | 8 | 8 | 0.5 | 1.063 | IND |
|  | MRSA 7113 | 4 | 4 | 0.125 | 4 | 1.031 | IND |
| **Kuwanon C** | MRSA 7109 | 2 | 16 | 0.125 | 8 | 0.563 | PSYN |
|  | MRSA 7110 | 4 | 16 | 0.25 | 8 | 0.563 | PSYN |
|  | MRSA 7112 | 4 | 8 | 4 | 0.5 | 1.063 | IND |
|  | MRSA 7113 | 2 | 4 | 0.0625 | 4 | 1.031 | IND |
| **Morusin** | MRSA 7109 | 4 | 16 | 2 | 4 | 0.75 | PSYN |
|  | MRSA 7110 | 8 | 16 | 0.25 | 8 | 0.531 | PSYN |
|  | MRSA 7112 | 4 | 8 | 2 | 4 | 1 | PSYN |
|  | MRSA 7113 | 4 | 4 | 2 | 2 | 1 | PSYN |
| **Kuwanon H** | MRSA 7109 | 2 | 16 | 2 | 0.5 | 1.031 | IND |
|  | MRSA 7110 | 4 | 16 | 0.25 | 8 | 0.563 | PSYN |
|  | MRSA 7112 | 2 | 8 | 1 | 4 | 1 | PSYN |
|  | MRSA 7113 | 2 | 4 | 0.125 | 4 | 1.063 | IND |
| **Albafuran C** | MRSA 7109 | 8 | 16 | 2 | 8 | 0.75 | PSYN |
|  | MRSA 7110 | 8 | 16 | 2 | 8 | 0.75 | PSYN |
|  | MRSA 7112 | 8 | 8 | 0.25 | 8 | 1.031 | IND |
|  | MRSA 7113 | 8 | 4 | 0.25 | 4 | 1.031 | IND |

ATB, antibiotic; comb, in combination; FICi, fractional inhibitory concentration index; IND, indifference; MIC, minimum inhibitory concentration in µg/ml; MRSA, methicillin-resistant *Staphylococcus aureus*; NC, natural compound; PSYN, partial synergism
